# Comparison of Single and Combined Use of Catechin, Protocatechuic, and Vanillic Acids as Antioxidant and Antibacterial Agents against Uropathogenic *Escherichia Coli* at Planktonic and Biofilm Levels

**DOI:** 10.3390/molecules23112813

**Published:** 2018-10-30

**Authors:** Ariadna Thalia Bernal-Mercado, Francisco Javier Vazquez-Armenta, Melvin R. Tapia-Rodriguez, Maria A. Islas-Osuna, Veronica Mata-Haro, Gustavo A. Gonzalez-Aguilar, Alonso A. Lopez-Zavala, Jesus Fernando Ayala-Zavala

**Affiliations:** 1 Centro de Investigacion en Alimentacion y Desarrollo, AC, Carretera a la Victoria km. 0.6, Hermosillo 83000, Mexico; thalia.bernal@estudiantes.ciad.mx (A.T.B.-M.); javier.vazquez@estudiantes.ciad.mx (F.J.V.-A.); melvin.tapia@estudiantes.ciad.mx (M.R.T.-R.); islasosu@ciad.mx (M.A.I.-O.); vmata@ciad.mx (V.M.-H.); gustavo@ciad.mx (G.A.G.-A.); 2 Universidad de Sonora, Blvd. Luis Encinas y Rosales S/N, Col. Centro, Hermosillo, Sonora 83000, Mexico; alexis.lopez@unison.mx

**Keywords:** synergism, phenolic compounds, antimicrobials, anti-biofilm compounds, antibiotic resistance, bacterial adhesion, urinary catheter infection

## Abstract

The objective of this study was to evaluate the effect of combining catechin, protocatechuic, and vanillic acids against planktonic growing, adhesion, and biofilm eradication of uropathogenic *Escherichia coli* (UPEC), as well as antioxidant agents. The minimum inhibitory concentrations (MIC) of protocatechuic, vanillic acids and catechin against the growth of planktonic bacteria were 12.98, 11.80, and 13.78 mM, respectively. Mixing 1.62 mM protocatechuic acid + 0.74 mM vanillic acid + 0.05 mM catechin resulted in a synergistic effect acting as an MIC. Similarly, the minimum concentrations of phenolic compounds to prevent UPEC adhesion and biofilm formation (MBIC) were 11.03 and 7.13 mM of protocatechuic and vanillic acids, respectively, whereas no MBIC of catechin was found. However, combinations of 1.62 mM protocatechuic acid + 0.74 mM vanillic acid + 0.05 mM catechin showed a synergistic effect acting as MBIC. On the other hand, the minimum concentrations to eradicate biofilms (MBEC) were 25.95 and 23.78 mM, respectively. The combination of 3.20 mM protocatechuic acid, 2.97 mM vanillic acid, and 1.72 mM catechin eradicated pre-formed biofilms. The antioxidant capacity of the combination of phenolics was higher than the expected theoretical values, indicating synergism by the DPPH^•^, ABTS, and FRAP assays. Effective concentrations of catechin, protocatechuic, and vanillic acids were reduced from 8 to 1378 times when combined. In contrast, the antibiotic nitrofurantoin was not effective in eradicating biofilms from silicone surfaces. In conclusion, the mixture of phenolic compounds was more effective in preventing cell adhesion and eradicating pre-formed biofilms of uropathogenic *E. coli* than single compounds and nitrofurantoin, and showed antioxidant synergy.

## 1. Introduction

Catheter-associated urinary tract infection (CA-UTI) is commonly acquired in hospitals around the world [1]. This infection is caused by uropathogenic *E. coli* (UPEC) due to its capacity to adhere to catheters and develop biofilms [2,3]. UPEC biofilms on catheters include communities of microorganisms adhered to a silicon surface, embedded in an extracellular polymeric substances matrix, and with altered metabolism compared to the corresponding planktonic cells [4]. The biofilm-secreted polymeric substances protect the embedded cells against antibiotics, evade the host immune defense, and promote persistence in the environment, causing recurrent infections [4]. In addition, persistent UTIs can cause pyelonephritis, leading to parenchymal injury or renal scarring, activation of inflammatory mediators, and overproduction of reactive oxygen species [5]. Consequently, the treatment of UTI is a significant challenge, considering bacterial evolution against conventional treatments, mainly when resistance at the planktonic cellular level and community level occur; besides, the oxidative complications involved with this infection [6].

UTI is commonly treated with antibiotics, such as ampicillin, trimethoprim, cephalosporin, nalidixic acid, and nitrofurantoin [7], but alternatives need to be evaluated, considering the rapid emergence of antibiotic resistance, the presence of inflammation, and oxidative damage. It has been reported that planktonic *E. coli* isolated from the urine of patients with CA-UTI are resistant to most frequently used antibiotics [3]. Antibiotics are developed to inhibit bacterial growth or kill bacteria in planktonic cells, but these agents are less active in avoiding cellular adhesion, inactivate formed biofilms, or inactivate free radicals [8]. The recurrence of UTI, bacterial biofilms resistance, and the oxidative damage of the tissue have promoted the search for alternative antimicrobial-antioxidant therapies [9,10]. 

The consumption of functional plant foods and medicinal plants (for example, cranberry juice rich in proanthocyanidins) has been widely recommended to prevent urinary infections [9]. However, after the consumption of cranberry, the complex phenolic compounds are metabolized to simple phenols like phenolic acids and flavonoids [11]. Several clinical studies have reported the presence of these compounds in urine after cranberry ingestion, with protocatechuic acid, vanillic acid, and catechin as the most commonly found [12,13]. These results suggest that those specific phenolic compounds could also exert an antibacterial effect, reduce urinary infections, and also act as antioxidants. Thus, the research question of this study is, what type of effect is caused by the combined presence of catechin and vanillic and protocatechuic acid on the growing, adhesion, and biofilm eradication of UPEC, as well as on the antioxidant capacity? The antibacterial potential of catechin, protocatechuic, and vanillic acids against planktonic *E. coli* has been tested and proved to be effective [14,15,16]; however, their efficacy to inhibit biofilm formation of uropathogenic *E. coli* has not been investigated, nor the effect of their ternary combination. The presence of these phenolic compounds could exert a synergic effect to control uropathogenic *E. coli* at different levels, including targeting planktonic cell survival, adhesion, biofilm eradication, and free radical inactivation [17]. In this context, the purpose of this study was to determine the effect of catechin, vanillic, and protocatechuic acids and their combination to prevent and eradicate uropathogenic *E. coli* biofilm on silicone catheters, besides acting as antioxidants.

## 2. Results

### 2.1. Antibacterial Activity of Individual and Combined Phenolic Compounds against Planktonic UPEC

Table 1 shows the effect of the individual use of phenolic compounds on the growth of planktonic UPEC cells. The minimum inhibitory concentration (MIC) of vanillic acid against bacterial growth was 11.80 mM, and the minimum bactericidal concentration (MBC) was 17.84 mM. Similarly, 12.98 mM of protocatechuic acid was needed to inhibit uropathogenic bacteria growth, while 19.46 mM was used to cause bacterial death. The MIC of catechin was 13.78 mM, and its bactericidal concentration was not found at the tested range (1.72–34.45 mM). On the other hand, the MIC and MBC of nitrofurantoin were 0.4 mM. Favorably, a synergistic interaction was obtained when combining 1.62 mM protocatechuic acid, 0.74 mM vanillic acid, and 0.05 mM catechin with a ΣFIC of 0.3 (Table 2); meanwhile, other combinations showed additive or indifferent interactions. It is important to note that these concentrations were selected based on the criteria of the lowest doses of the ternary combination with higher antibacterial efficacy by using the checkerboard method. However, it is important to mention that other effective ternary combinations could be found with different doses of the active compounds. These results reflected the impact of combining phenolics to reduce effective doses and helped define a range of concentrations to be used in the antibiofilm assays, discarding losses in planktonic cellular viability.

### 2.2. Minimum Inhibitory Concentrations of Individual and Combined Phenolic Compounds against Uropathogenic E. coli Adhesion on Silicone Urinary Catheters

**Figure 1:**
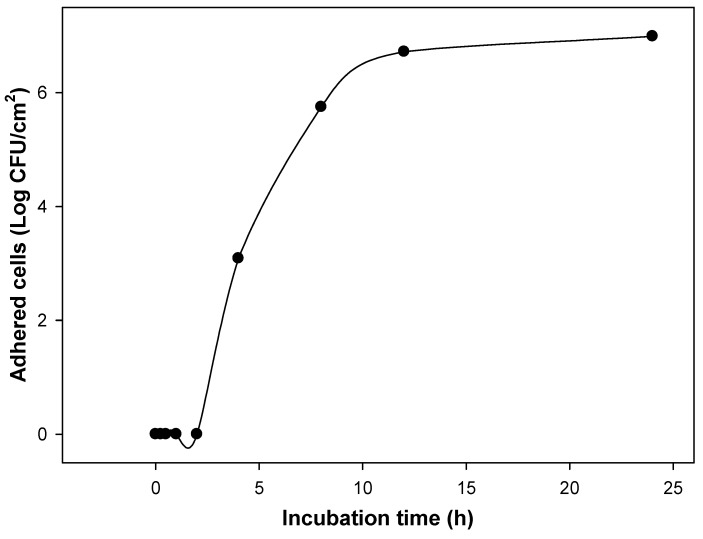
Uropathogenic *E. coli* adhesion on silicone catheter surfaces for 24 h at 37 °C. A significant effect of the incubation time on the cellular adhesion was found (*p* ≤ 0.05). Means for three independent experiments. The initial inoculum added to the system was 3 Log CFU/mL.

**Figure 2:**
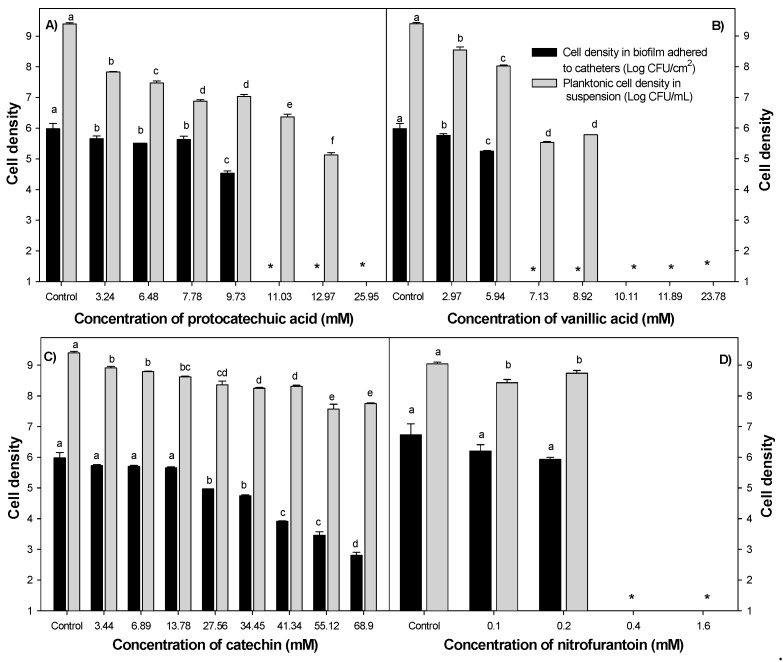
Effect of (**A**) protocatechuic acid, (**B**) vanillic acid, (**C**) catechin, and (**D**) nitrofurantoin on uropathogenic *E. coli* cell density in biofilms adhered to catheters (Log CFU/cm^2^) and planktonic cells (Log CFU/mL) for 24 h at 37 °C. Different letters indicate significant differences among concentrations of phenolic compounds at the same cellular level (*p* ≤ 0.05). The initial inoculum added to the system was 3 Log CFU/mL. Means for three independent experiments ± deviation standard are illustrated. * Mean values below the limit of detection.

*E. coli* adhesion on silicone catheters was detected at the 4th hour of incubation at 37 °C (Figure 1), reaching a plateau on viable adhered cells at 12 h with no change up to 24 h, indicating the short time required to start cellular adhesion and that a mature biofilm is formed at 24 h. The activity of individual phenolic compounds and nitrofurantoin against adhered and planktonic uropathogenic *E. coli* revealed that phenolic compounds affected the cellular adhesion by other means that only affect the viability of the added planktonic inoculum, as was done by nitrofurantoin (Figure 2). Protocatechuic acid slightly reduced bacterial biofilms at concentrations of 3.24–7.78 mM compared to bacteria without phenolic exposition (*p* ≤ 0.05) (Figure 2A). However, bacterial adhesion was inhibited entirely at concentrations higher than 11.03 mM, and it still showed viable planktonic cells, remembering that the initially added inoculum was 3 Log CFU/mL. Therefore, 11.03 mM was selected as the minimum inhibitory concentration of protocatechuic acid against biofilm formation. Similar results were observed using vanillic acid (Figure 2B); its minimum biofilm inhibitory concentration (MBIC) was 7.13 mM, and at this concentration, the planktonic cell growth was not impaired compared to the initially added inoculum (*p* ≤ 0.05). Besides, lower concentrations than 7.13 mM were also sufficient to reduce adhered cells to silicone surfaces without affecting planktonic growth, and higher doses than 10.11 mM inhibited planktonic and biofilm cells. Regarding catechin (Figure 2C), it reduced attached cells in a dose-dependent way; however, its MBIC was not determined at the tested doses. Besides, planktonic cells were slightly affected by catechin at 68.90 mM, suggesting that it had no bactericidal effect but still reduced bacterial adhesion. By contrast, nitrofurantoin at 0.4 mM inhibited bacterial biofilm formation as a consequence of cell death. This can be assumed by analyzing the effect of lower doses where no effect of this antibiotic against adhered or planktonic cells was observed (Figure 2D). These results are significant, considering that they indicated an affection of the cell adhesion mechanisms by phenolic compounds, and a planktonic cell is more sensitive to environmental distresses.

**Figure 3:**
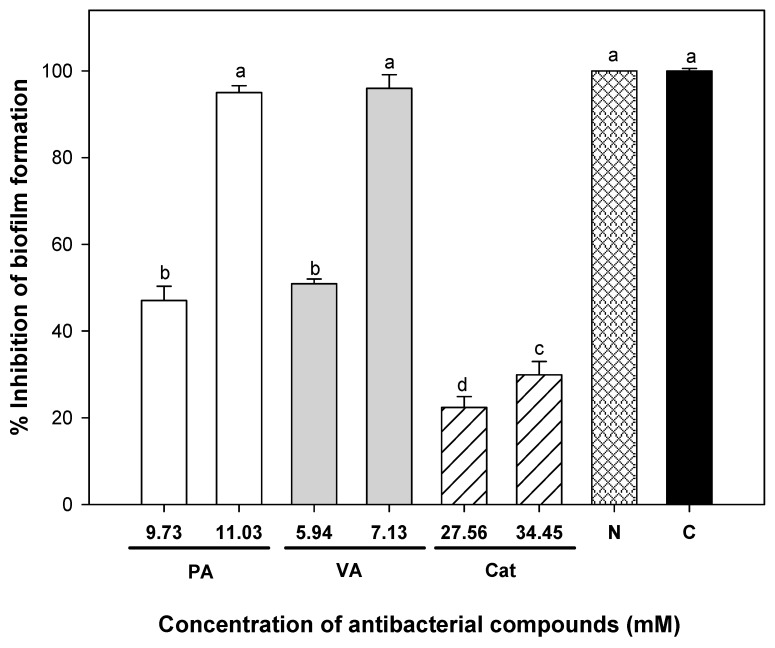
Percentage of the inhibition of uropathogenic *E. coli* biofilm formation incubated for 24 h at 37 °C in the presence of single and combined phenolic compounds determined by crystal violet assay. Means for three independent experiments ± deviation standard are illustrated. PA = protocatechuic acid, VA = vanillic acid, Cat = catechin, N = nitrofurantoin 0.4 mM, C = Combination (1.62 mM protocatechuic acid, 0.74 mM vanillic acid, and 0.05 mM catechin). Different letters indicate significant differences among treatments (*p* ≤ 0.05). The initial inoculum added to the system was 3 Log CFU/mL.

Subsequently, different concentrations of phenolic compounds were mixed and evaluated on the adhesion of *E. coli* cells to silicone surfaces; Table 3 shows the interaction effects of some of these combinations. Among these mixtures, three were effective to inhibit utterly bacterial adhesion on catheters without inhibiting the viability of planktonic cells in comparison to the initially added inoculum. One of these combinations showed a synergistic effect with 1.62 mM protocatechuic acid + 0.74 mM vanillic acid + 0.05 mM catechin. At this point, reductions of 6.89, 9.63, and 1398 times of protocatechuic acid, vanillic acid, and catechin were achieved when combining the tested phenolic compounds in comparison to effective doses of individual compounds to avoid bacterial adhesion. A similar pattern was found using the crystal violet assay to evaluate the effects of the tested compounds on the biomass presence over polystyrene surfaces of microplate wells (Figure 3). The most active compound to reduce biomass was vanillic acid at 7.13 mM; also, bacteria exposed to 11.03 mM protocatechuic acid showed lower crystal violet staining, while a maximum of 30% of biofilm inhibition was recorded for catechin at 68.9 mM. In comparison, nitrofurantoin displayed 100% inhibition of biofilm formation, as well as the phenolic combination previously found to avoid cell adhesion to silicone catheters (1.62 mM protocatechuic acid + 0.74 mM vanillic acid + 0.05 mM catechin).

### 2.3. Effect of Individual and Combined Phenolic Compounds to Eradicate Pre-Formed Uropathogenic E. coli Biofilms

**Figure 4:**
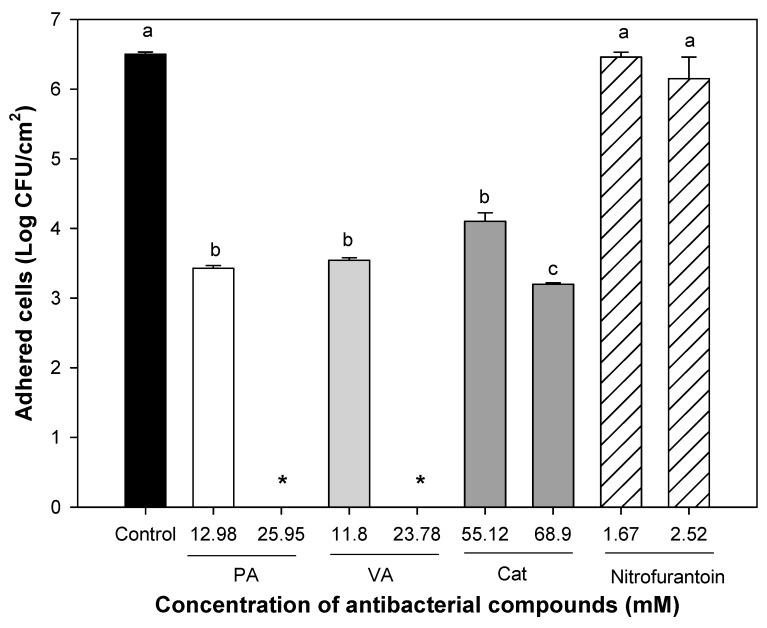
Viability of uropathogenic *E. coli* in pre-formed biofilms exposed during 1 h to phenolic compounds and nitrofurantoin. PA = protocatechuic acid, VA = vanillic acid, and Cat = catechin. Different letters indicate significant differences among treatments (*p* ≤ 0.05). The means for three independent experiments ± deviation standard are illustrated in the table. * Mean values below the detection limit.

Phenolic acids were effective in reducing the number of viable cells from pre-formed biofilms of uropathogenic *E. coli* attached to silicone surfaces (Figure 4). The MIC of protocatechuic acid reduced cell density in biofilms, but did not completely eradicate them (*p* ≤ 0.05); however, increasing its MIC two times (25.95 mM) was sufficient to eradicate UPEC biofilms. Regarding the effect of vanillic acid, a concentration of 23.78 mM (twice the MIC) was effective, inactivating 100% of viable cells within pre-formed biofilms (*p* ≤ 0.05). In the case of catechin, both tested concentrations (55.12 and 68.9 mM) were effective to reduce cell density in mature biofilms (*p* ≤ 0.05), but no minimum eradication dose was found, which is consistent with our previous assays where MBC was not determined. In contrast, nitrofurantoin was not active against bacteria embedded in pre-formed biofilm on silicone surfaces at the tested concentrations (0.2–2.5 mM). These results reflected the interference offered by biofilms against the action of antibacterial agents; besides, they reflected that nitrofurantoin was not useful to attack the viability of bacteria within pre-formed biofilms. The effect of combined phenolic compounds on biofilm eradication is shown in Table 4. Three synergistic combinations were found, but the combination of 3.2 mM protocatechuic acid + 2.97 mM vanillic acid + 1.72 mM catechin showed the lowest concentrations of each compound that inactivated 100% of bacteria embedded in pre-formed biofilms.

**Figure 5:**
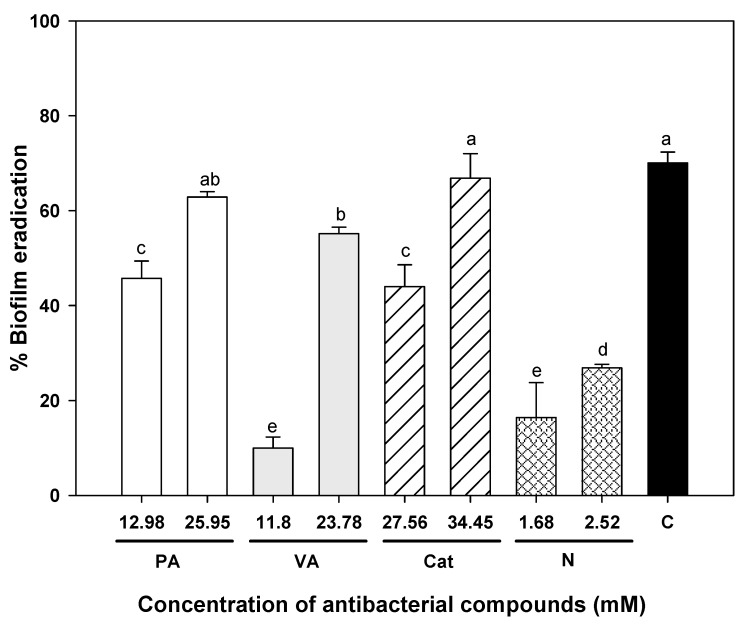
Percentage of eradication of pre-formed uropathogenic *E. coli* biofilms incubated for 24 h at 37 °C and exposed to single and combined presence of phenolic compounds for 1 h. Means for three independent experiments ± deviation standard are illustrated. PA = protocatechuic acid, VA = vanillic acid, Cat = catechin, N = nitrofurantoin, C = Combination (1.62 mM protocatechuic acid, 0.74 mM vanillic acid and 0.05 mM catechin). Different letters indicate significant differences among treatments (*p* ≤ 0.05).

Phenolic compounds were also able to decrease the biomass of pre-formed UPEC biofilms by the crystal violet assay (Figure 5). Biofilms exposed to 25.95 mM protocatechuic acid showed reduced crystal violet staining, showing only 37% of adhered biomass compared to the control biofilm. In contrast with other results, catechin at 34.45 mM was the most effective of all the individual compounds at decreasing biofilm biomass, showing a 67% reduction; however vanillic acid at lower doses (23.78 mM) induced an eradication of UPEC biofilm biomass of up to 55%. Nitrofurantoin at 2.52 mM only was able to eradicate 27% of UPEC biomass compared to the control biofilm. The combination of 3.20 mM protocatechuic acid, 2.97 mM vanillic acid, and 1.72 mM catechin eradicated 70% of bacterial biofilm, showing the highest inhibition with the lowest doses. These results confirmed that treatment with nitrofurantoin only is effective against UPEC at the planktonic level.

### 2.4. Antioxidant Activity of Individual and Combined Phenolic Compounds

Results confirmed that individual phenolic compounds possess high antioxidant capacity, and it depends on their molecular structure and evaluation assay. The antioxidant capacity values of catechin were higher than the values observed for vanillic and protocatechuic acids (Table 5). Theoretical antioxidant values of the phenolic combinations were obtained adding the individual antioxidant capacity values of each compound. Then, the differences between the experimental and theoretical antioxidant values were obtained to visualize synergism (difference with positive values) or antagonism (difference with negative values) (Table 6). The combinations tested were those indicated by the MIC/MBIC (1.62 mM protocatechuic acid + 0.74 mM vanillic acid + 0.05 mM catechin) and the MBEC (3.20 mM protocatechuic acid + 2.97 mM vanillic acid + 1.72 mM catechin). The MIC/MBIC combination showed synergistic effects in all the assays. Regarding the MBEC combination, a synergistic effect of the combination was found in testing its antioxidant capacity by the FRAP and DPPH assays, while the antagonistic effect was found using the ABTS^•+^ assay.

## 3. Discussion

The tested phenolic compounds were useful to inhibit the growth of planktonic cells and cause bacterial death. There is evidence that phenolic acids can cause irreversible modifications to the permeability of bacterial membranes and decrease cell surface charges, causing rupture or forming pores, with consequent leakage of intracellular components [18]. In addition, flavonoids, like catechin, could chelate metals essentials as enzymatic cofactors involved in bacterial growth [19]. These different targets for the tested phenolic compounds could be the reason for the observed synergy. It has to be noticed that even when catechin showed no bactericidal effect at the highest tested concentrations, it seems to have a role in improving the efficacy of phenolic acids in the combination. 

No previous studies have reported the antibacterial effect of these phenolic acids and catechin against UPEC; however, similar MICs have been reported for these individual phenolic acids against other bacteria [15,20]. Protocatechuic acid showed an MIC of 19.46 mM against *Listeria monocytogenes,* and vanillic acid showed MICs of 17.84 and 14.86 mM against *E. coli* O157:H7 and *Salmonella* Typhimurium, respectively. On the other hand, flavonoids such as catechins, showed antibacterial potential with MICs ranging from 31 mM to 344 mM against *E. coli*, *S*. Typhimurium, and *Staphylococcus aureus* [16,21]. In average, the obtained MIC values are in the range previously reported, and the found variations could be attributed to different bacterial strains.

Adhesion of uropathogenic *E. coli* on the silicone surface of catheters is responsible for its persistence in hospital environments. The inhibition of adhesion is the first step in interrupting biofilm formation and its complications, including UTI and pyelonephritis. Regrettably, few antibiotics have this ability, including nitrofurantoin, and the observed anti-adhesive effect of protocatechuic and vanillic acids must be recognized, as well as the eradication of pre-formed biofilms and contribution of catechin to antioxidant capacity. Trying to relate the observed effect of the tested phenolic compounds with their molecular properties, it has to be mentioned that vanillic acid is more lipophilic (log *p* = 1.43) than protocatechuic acid (Log *p* = 0.86), and catechin (Log *p* = 0.4), and, because of this, it could easily interfere with the cell membrane and possibly affect bacterial adhesion. Previous reports indicated that gallic, ferulic, caffeic, and chlorogenic acids prevented bacterial adhesion, increased superficial hydrophobicity, and decreased the cell adhesion potential [22,23,24]. Furthermore, bacterial adhesion can be affected by inhibiting the secretion of polymeric substances [25]. Epigallocatechin gallate can eliminate biofilm matrix of *E. coli* by interfering with the assembly of curli subunits into amyloid fibers and cellulose biosynthesis, and by reducing the expression of CsgD—a crucial activator of curli and cellulose [25].

Phenolic compounds can interfere with cell-cell communication by various mechanisms, such as inhibiting the synthesis of signal molecules or receptors, which in turn affects the synthesis of polymeric extracellular substances and biofilm development [26]. It is known that compounds like quercetin, kaempferol, naringenin, and apigenin reduced *E. coli* biofilm formation because they suppressed the autoinducer-2 activity, which is responsible for cell-to-cell communication involved in biofilm production [26]. In addition, Lee et al. [27] confirmed that phloretin belonging to flavonoids suppressed autoinducer-2 importer genes of *E. coli* O157:H7 biofilm cells, confirming the inhibition of cell communication. Thus, it would be interesting to test whether some of these mechanisms are involved in the observed UPEC responses caused by phenolic compounds. 

The achieved dose reductions when combining phenolic compounds were 6.89, 9.63, and 1398 times for protocatechuic acid, vanillic acid, and catechin against UPEC adhesion, respectively. At this point, the effective doses to avoid cell adhesion were lower than those needed to have a bactericidal effect. Another interesting fact is that nitrofurantoin did not seem to affect any mechanism of adhesion but instead attacked the viability of planktonic cells; this could be attributed to its mode of action. It does not interfere with motility, adhesion, or cellular communication, which are factors influencing biofilm development [28]. However, nitrofurantoin’s mechanism of action is complex and not completely understood, but it appears to be caused by the production of nitrofurans (reactive intermediates) that can damage ribosomal proteins, DNA, and other macromolecules [29]. For this reason, it is important to consider alternative antibacterial agents to this antibiotic. Besides, as observed previously, cell adhesion and biofilm formation of UPEC took a short time (4 h) in catheters (Figure 1), and nitrofurantoin only affected planktonic cells. Its administration must occur before cellular attachment does.

Higher doses of phenolic compounds were needed to eradicate biofilms compared to planktonic cell adhesion, and this reflects the attributed resistance granted to this association against the tested antimicrobial agents. However, vanillic and protocatechuic acids were effective at eliminating viable cells from 24 h pre-formed biofilms of uropathogenic *E. coli* on silicone surfaces; while catechin seemed to disrupt the biofilm biomass. The achieved reductions combining phenolic compounds were 8, 8, and 40 times for protocatechuic acid, vanillic acid, and catechin, respectively. No reduction of viable cells from pre-formed biofilms was achieved using nitrofurantoin, and these results reinforced the need for using alternatives to prevent biofilm formation and substitutes for this antibiotic. It has been reported that biofilm removal and biofilm cell inactivation are done by distinct processes. Biofilm eradication refers to inactivating bacteria, but those can remain attached to the surface; on the other hand, biofilm removal has to be done by the elimination of the attached extracellular polymeric substances. Any agent with biofilm eradication activity must pass the matrix created by extracellular polymeric substances and attack the embedded viable cells. Biofilm bacteria can be inactivated by the antimicrobial activity of phenolic acids, which include disruption of cytoplasmic membranes, enzyme inhibition through reaction with the sulfhydryl group, or non-specific interactions with proteins and synthesis of nucleic acids [18]. Nevertheless, the mechanism in embedded biofilm bacteria could be different, considering the interference of the biofilm constituents. These results highlight the importance of combining antibacterial agents with a different mode of action, with at least one able to disrupt the polymer matrix and another able to cause bacterial cell death.

The antibacterial synergism observed in the combination of phenolic compounds could be attributed to different antibacterial mechanisms to inhibit biofilm formation. A possible synergistic mechanism proposed in our study is that vanillic acid and protocatechuic acid interact with the lipid layer of the bacterial membrane, affecting its structural integrity or functionality, and as a consequence, affect bacterial adhesion. This could facilitate the entrance of catechin, and this compound can interact with other molecules such as enzymes within the cytoplasm, affecting bacterial metabolism. The simultaneous action of these two mechanisms could be responsible for the observed synergistic antibacterial effect that inhibits cell density in biofilm formation, but more research is needed to test this hypothesis. 

It was observed that the phenolic combinations used against planktonic and biofilm inhibition had a synergistic effect, and their combination at the used proportions in the biofilm eradication test had a synergistic effect in the DPPH and FRAP assays. The difference in the results observed between the used reactive specie could be attributed to their different reaction mechanism. Due to the complexity of simulating the characteristics of an oxidative reaction, it has been widely reported that multiple methods are necessary to test antioxidant activity [30]. Phenolic compounds are considered to be efficient hydrogen donors due to their number, arrangement of hydroxyl groups, and the specific carboxyl group, which can be easily ionized. A positive correlation has been found between the number of hydroxyl groups and antioxidant activity [31]. This molecular attribute could be the reason that catechin with five hydroxyls was the most effective antioxidant, followed by protocatechuic with two. Vanillic acid was the least antioxidant with only one hydroxyl group.

Furthermore, antioxidants can interact with each other when combined, showing some effects as synergistic, additive, and antagonistic. Some authors consider that a synergistic effect occurs when two or more antioxidants show a total effect higher than the sum of the individual effects. An additive effect refers to the sum of the effects of each compound, while an antagonistic effect reflects a lower effectiveness than the sum of the individual effects [32]. There could be three different mechanisms in the synergistic effect: 1. one compound makes the antioxidant effect, and the other regenerates it to have that effect again; 2. sacrificial oxidation of an antioxidant to protect the other; and 3. combination of two or more agents with different antioxidant mechanisms. 

Our results suggest that the studied phenolic compounds acted synergistically, depending on their concentration and the testing method, and they were able to donate hydrogen or transfer electrons to the reactive species and also regenerate or protect their partner compounds. Similar antioxidant capacities have been reported for some of these phenolic compounds: Palafox-Carlos, Gil-Chavez, Sotelo-Mundo, Namiesnik, Gorinstein and Gonzalez-Aguilar [31] reported that protocatechuic acid showed higher antioxidant capacity than vanillic acid against DPPH^•^ radical inhibition. In addition, these authors reported several binary, ternary, and quaternary combinations of phenolic acids, and most of these combinations had a synergistic effect. In addition, Skroza, Mekinic, Svilovic, Simar and Katalinic [30] investigated the antioxidant activity of phenolic binary mixtures. Their results suggested that some differences in the activity of the mixture were found depending on the method used. They demonstrated that the combination of catechin with resveratrol had a synergistic effect by DPPH^•^ and FRAP assays and that caffeic acid and resveratrol mixture showed synergistic activity by FRAP method, having an antagonistic effect by DPPH^•^ method. 

As stated before, persistent UTIs can cause pyelonephritis and overproduction of free radicals [33]; then, the addition of antioxidant compounds in its treatment is an alternative to relieving the symptoms caused by reactive oxygen species [5,10,34]. In this context, treatments with caffeic acid phenethyl ester (0.01 mM) were related to the repair of renal tissue damage after pyelonephritis, in addition to reducing chronic inflammation by enforcing the enzymatic antioxidant defense system [35]. Caffeic acid phenethyl ester administrated at 10 µM/kg body-weight reduced malondialdehyde and nitric oxide levels as well as xanthine oxidase activity, although it increased superoxide dismutase and glutathione peroxidase activities during pyelonephritis caused by *E. coli* in rats. In addition, a single dose of cranberry beverages with 111 g of phenolic compounds improved indices of oxidative stress, such as elevating blood glutathione peroxidase and superoxide dismutase activity, and exerted an ex vivo anti-adhesion activity against P-fimbriated *E. coli* compared to placebo in healthy humans, given evidence of the efficacy of the use of the therapeutic effect of extracts rich in phenolic compounds [36]. However, more information regarding the contribution of the studied compounds is needed. In summary, our results suggested that the combination of protocatechuic acid, vanillic acid, and catechin could be a promising alternative to use in UTI treatment; however, this study could be taken as the initial step to continue searching for more evidence to sustain the real impact of the alleged responses.

## 4. Materials and Methods

### 4.1. Antibacterial Effect of Individual Phenolic Compounds Against Planktonic Bacteria

Antibacterial activity of catechin, protocatechuic, and vanillic acids (≥97% HPLC, Sigma-Aldrich, Toluca, TOL, Mexico) was evaluated against uropathogenic *E. coli* (ATCC 700416) by the micro-well dilution assay method [37]. The inoculum was prepared using a 19 h-culture adjusted in Luria-Bertani (LB) broth to 1 × 10^5^ CFU/mL. Then, three µL of the adjusted inoculum were added to a sterile 96-well microtitre plate (Costar 96) to achieve a final concentration of inoculum of 1 × 10^3^ CFU/mL, followed by 297 µL of phenolic compounds at different concentrations (0–65 mM) diluted in LB broth with 5% of DMSO to dissolve and have a homogenous solution. The microplate was incubated at 37 °C for 24 h. The minimum inhibitory concentration (MIC) value of each phenolic compound was determined visually, according to the lowest concentration at which the tested bacteria did not show turbidity [38]. In addition, the minimal bactericidal concentration (MBC) of each compound was obtained by inoculating the MIC and three higher concentrations in LB agar and incubating at 37 °C for 24 h. Both MIC and MBC were expressed as millimolar (mM). All determinations were made in triplicates. The commercial antibiotic nitrofurantoin (Biofurin, Bioresearch de Mexico) was used as a positive control against uropathogenic *E. coli*, and, MIC and MBC were also determined.

### 4.2. Antibacterial Effect of Combined Phenolic Compounds against Planktonic Bacteria

The checkerboard method was followed to determine the effect of the ternary combination of phenolic compounds on their antibacterial effect. This method compares the antibacterial efficacy of combinations of the tested compounds at different ratios with their individual activity. First, binary mixtures were tested (protocatechuic acid + vanillic acid, vanillic acid + catechin, and catechin + protocatechuic acid), combining the following fractions of their MIC values (0.06, 0.125, 0.25, 0.5, 1, 2 × MIC). The fractional inhibitory concentration (ΣFIC) was used to determine the effect of combining phenolic compounds on their antibacterial efficacy compared to treatments with individual compounds [39]. This concentration was the addition of the antibacterial effect of each phenolic compound, and it was calculated as follows:

$$\Sigma FIC=\frac{MIC_{A}combination}{MIC_{A}\;individual}+\;\frac{MIC_{B}combination}{MIC_{B}\;individual}+\;\frac{MIC_{C}combination}{MIC_{C}\;individual}\;$$

The possible interactions between compounds in the mixture are according to ΣFIC values: synergistic, ΣFIC ≤ 0.5; additive, 0.5 < ΣFIC ≤ 1; indifferent, 1< ΣFIC ≤ 4; antagonistic, ΣFIC > 4.

The most effective binary combination (regarding antibacterial activity with the lowest doses) was selected to combine with the third compound. In ternary mixtures, those combinations that inhibit bacterial growth and presented an additive or synergistic effect were shown in the Table 4. The most effective ternary combination with the lowest doses and with the highest activity was selected as the synergistic combinations.

### 4.3. The Minimum Inhibitory Concentrations of Phenolic Compounds against Biofilm Formation of Uropathogenic E. coli

The needed time to form mature biofilms on silicone catheters was determined before testing the selected phenolic compounds. Cylindrical fragments of 1 cm from a sterile Foley catheter (24 Fr/Ch, 8 mm) were used as a silicone surface because of its recurrent use in clinical applications. Under sterile conditions, the Foley catheter fragments were placed inside glass tubes containing five mL of LB broth. The inoculums were prepared from cultures growing in the exponential phase (19 h at 37 °C in LB, Figure S1) and were adjusted in LB tubes at 10^3^ CFU/mL. During different incubation times at 37 °C, the catheter fragments were removed and washed with sterile distilled water to eliminate non-adherent cells. Immediately after, catheter fragments were placed in 5 mL of saline solution and were subjected to an ultrasonic bath (40 kHz) for 5 min in order to detach the adherent cells. From the bacterial suspension obtained after sonication, serial dilutions were carried out to determine the number of adherent bacteria per unit of area (log CFU/cm^2^) using LB agar and incubated for 24 h at 37 °C [40]. After this, the effect of different concentrations of individual and combined phenolic compounds on viable adhered cells of UPEC to catheter silicone surfaces was evaluated as described above. Individual phenolic compounds’ solutions were added and incubated at 37 °C for 24 h. Changes of adherent and suspended UPEC cells were determined to compare untreated and treated bacteria. Planktonic cells in the initial suspension were counted in LB agar after 24 h of incubation at 37 °C to ensure that compounds affect bacterial adhesion without affecting the viability of the added inoculum. The lowest concentration of each compound to inhibit the attachment of bacteria was defined as the MBIC [24]. Moreover, the MBIC of nitrofurantoin was also determined to evaluate its effect on bacterial biofilm. Three independent experiments were performed.

For testing the phenolic mixtures against cellular adhesion, an adaptation of the checkerboard was followed, with the same criteria of highest efficacy with the lowest doses. First, binary mixtures were made based on MBIC of each compound. Then, the mixture with the best effect was combined with the third compound. Subsequently, these combinations were added to tubes containing the silicone fragment and the bacterial inoculum, and the same procedure as described for individual compounds was followed.

### 4.4. The Minimum Inhibitory Concentrations of Phenolic Compounds to Eradicate Pre-Formed Biofilms of Uropathogenic E. coli

Firstly, mature UPEC biofilms were pre-formed for 24 h at 37 °C on silicone surfaces as previously described in Section 4.3. After that time, catheter fragments were washed with sterile water and then exposed for one hour at the different individual (0, 0.5, 1, 2, and 3 times the MIC) and combined concentrations of catechin, protocatechuic and vanillic acids diluted in saline solution [41]. Then, catheters were washed, placed in saline solution, and sonicated (40 kHz) for 5 min. Afterward, serial dilutions were carried out to determine the survival number of bacteria per unit of area (Log CFU/cm^2^) using LB agar. The minimum biofilm eradicating concentration (MBEC) was determined as the concentration of phenolic compound that inactivated bacteria from a pre-formed biofilm on the silicon surface [41]. The MBEC of nitrofurantoin was also determined. Three independent experiments were performed.

### 4.5. Crystal Violet Assay to Evaluate the Inhibition and Eradication of Uropathogenic E. coli Biofilm by Exposure to Individual and Combined Phenolic Compounds

The effects of phenolic compounds, individual and combined, as well as nitrofurantoin on biofilm formation and eradication, were also followed using the crystal violet assay [42]. Bacterial inoculum was prepared at 1 × 10^3^ CFU/mL in LB broth from an exponential phase culture (18 h in LB broth). For the inhibition of biofilm formation assay, three µL of the bacterial solution and 297 µL of single and combined phenolic compound and nitrofurantoin at different concentrations were taken and placed in sterile 96-well polystyrene microplates (Costar 96). For the eradication test, only bacterial inoculum without any phenolic compound was added to the well. Then, both microplates were incubated for 24 h at 37 °C. For the eradication test, the bacterial culture medium with unattached cells was removed, and the microplate was gently washed with sterile distilled water; then, 300 µL of different concentrations of single and combined phenolic compounds were added to the microplate for 1 h. After that time, in both microplates (eradication and formation tests), the medium was removed by aspiration and washed three times with distilled water. Then, the microplates were allowed to dry for 15 min. Consequently, biofilms were stained with 150 µL of 0.1% (*w*/*v*) crystal violet for 45 min at room temperature. The unbound crystal violet dye was removed, and wells were washed gently three times with distilled water. The wells were dried for 15 min, and the crystal violet in each well was solubilized by adding 150 µL of 33% acetic acid for 15 min. Subsequently, 150 µL of sample was transferred to another well, and the OD was measured at 600 nm in a FLUOstar Omega spectrophotometer (BMGLabtech, Chicago, IL, USA). Each measure was performed in triplicate, and LB broth without any treatment was used as a blank. Results were expressed as the percentage of reduction of biomass produced (OD), considering as 100% the value of the control biomass bacterial.

### 4.6. Antioxidant Capacity of Individual and Combined Phenolic Compounds Using the DPPH^•^, TEAC, and FRAP Assays

The antioxidant activity values of individual phenolic compounds were determined, and with their addition, the theoretical antioxidant values of their combinations were obtained using the DPPH^•^, ABTS^•+^, and FRAP assays. Then, the differences between the experimental and theoretical antioxidant values were used to visualize synergism (difference with positive values) or antagonism (difference with negative values). The tested combinations were those indicated by the MIC/MBIC (1.62 mM protocatechuic acid + 0.74 mM vanillic acid + 0.05 mM catechin) and the MBEC (3.20 mM protocatechuic acid + 2.97 mM vanillic acid + 1.72 mM catechin). 

#### 4.6.1. DPPH^•^ Radical Scavenging Activity

Antioxidant activity was determined using the 2,2-diphenyl-1-picrylhydrazyl (DPPH^•^) method [43]. In darkness, a stock solution was prepared to mix 2.5 mg of DPPH^•^ radical with 100 mL of pure methanol. The absorbance of the DPPH^•^ solution was adjusted to 0.70 measured at 515 nm using a FLUOstar Omega spectrophotometer. Then, 140 μL of the radical solution and 10 μL of each sample were mixed and incubated for 30 min, and absorbance was read at 515 nm. Trolox was used as a standard, and results were expressed as mmol of TE per mmol individual phenolic compounds, and the combined compounds’ results were expressed as μM of Trolox equivalents (the combinations were diluted 25 times to fit the Trolox standard curves).

#### 4.6.2. ABTS Radical Scavenging Activity

The Trolox-equivalent antioxidant capacity (TEAC) of the samples was calculated using ABTS^•+^ [2,2′-azino-bis(3-ethylbenzothiazoline-6-sulfonic acid)] [43]. The ABTS^•+^ radical was generated in darkness by mixing 5 mL of a solution of 7 mM ABTS^•+^ with 88 μL of 0.139 mM solution of K_2_S_2_O_8_. The radical solution was adjusted in methanol to an OD of 0.7 measured at 754 nm. For the assay, 5 μL of the sample, and 245 μL of the ABTS solution were mixed, and, after 6 min, OD was measured with a FLUOstar Omega spectrophotometer. Trolox was used as a standard, and the results were expressed as mmol of TE per mmol individual phenolic compounds; and for the combination, the results were expressed as μM of Trolox equivalents. (The combinations were diluted 25 times to fit the Trolox standard curves.)

#### 4.6.3. FRAP Assay 

The FRAP assay was carried out following the method reported by Velderrain-Rodriguez et al. [44]. Twenty μL of each sample were added to a microplate well and mixed with 280 μL of FRAP reagent. The samples were kept in darkness for 30 min at room temperature, and the absorbance was read at 593 nm in a FLUOstar Omega spectrophotometer. Trolox was used as a standard, and results were expressed as mmol of TE per mmol individual phenolic compounds, and for the combined compounds were expressed as μM of Trolox equivalents (the combinations were diluted 25 times to fit the Trolox standard curves).

### 4.7. Statistical Analysis

A completely randomized experimental design was applied to all experiments. Factors evaluated were the concentrations of catechin, protocatechuic, and vanillic acids, and the variable responses were the number of cells adhered to silicone (log CFU/cm^2^), the number of cells in the planktonic state (Log CFU/mL), the percentage of biofilm inhibition and eradication (%), and the mmol of Trolox equivalents/mmol in antioxidants tests. An analysis of variance (ANOVA) was performed to estimate significant differences between treatments, and the Tukey-Kramer test was applied to compare means (*p* ≤ 0.05) using the NCSS 2007 software (NCSS LLC, Kaysville, UT, USA). 

## 5. Conclusions

Combining protocatechuic acid with vanillic acid and catechin showed a synergistic effect on the inhibition of the growth of planktonic cells, biofilm formation, and eradication of uropathogenic *E. coli* on silicone surfaces. This study demonstrated the potential of combining phenolic compounds to increase antibiofilm efficacy and reduce effective doses. In addition, the antioxidant capacity of the studied compounds was improved with their combination, as observed with their ability to inactivate reactive species. Further studies could be performed in order to establish the mechanisms of each phenolic compound in the synergistic mixture.

## Figures and Tables

**Table 1:** Minimum inhibitory concentration (MIC) and minimum bactericidal concentration (MBC) of phenolic compounds against uropathogenic *E. coli* at the planktonic level.

Compounds	MIC (mM)	MBC (mM)
Protocatechuic acid	12.98	19.46
Vanillic acid	11.80	17.84
Catechin	13.78	ND
Nitrofurantoin	0.4	0.4

ND: not determined in the tested range 1.72–34.45 mM.

**Table 2:** Effect of combining phenolic compounds against planktonic cells of uropathogenic *E. coli.*

Phenolic Compound (mM)			Effect	
Protocatechuic Acid	Vanillic Acid	Catechin	ΣFIC	Interaction
0.81	5.94	0.0	0.5	Synergistic
12.98	0.0	0.20	1.0	Additive
0.0	5.94	6.89	1.0	Additive
1.62	0.17	6.89	0.6	Additive
1.62	0.74	0.05	0.3	Synergistic
6.48	2.97	3.44	1.0	Additive

**Table 3:** Effect of combined phenolic compounds on counts of adhered uropathogenic *E. coli* cells on silicone surfaces and planktonic cells (24 h at 37 °C).

Combination (mM)						
Protocatechuic Acid	Vanillic Acid	Catechin	Adhered Cells (Log CFU/cm^2^)	Planktonic Cells (Log CFU/mL)	ΣFIC	Combination Effect
0.0	0.0	0.0	5.96 ± 0.1	8.76 ± 0.01		
2.75	1.48	13.78	4.23 ± 0.0	8.59 ± 0.09	0.62	Additive
5.51	1.48	13.78	3.68 ± 0.17	8.47 ± 0.07	0.87	Additive
2.75	3.56	13.78	5.14 ± 0.04	8.35 ± 0.05	0.95	Additive
5.51	3.56	13.78	2.35 ± 0.05	3.08 ± 0.01	1.20	Indifferent
1.62	0.74	0.05	ND	8.78 ± 0.06	0.25	Synergistic
3.20	2.97	1.72	3.3 ± 0.13	8.71 ± 0.06	0.74	Additive
6.48	2.97	3.44	4.41 ± 0.06	7.03 ± 0.01	1.05	Indifferent
1.62	5.94	3.44	ND	6.37 ± 0.02	1.18	Indifferent
3.24	2.97	-	5.95 ± 0.07	8.86 ± 0.04	0.71	Additive
2.75	5.94	-	ND	7.02 ± 0.03	1.08	Indifferent
6.48	2.97	-	3.35 ± 0.1	8.02 ± 0.15	0.88	Additive
-	5.94	3.44	5.6 ± 0.1	8.14 ± 0.06	1.00	Indifferent

ND: not detected. Means for three independent experiments ± deviation standard. The initial inoculum added to the system was 3 Log CFU/mL.

**Table 4:** Combinations of phenolic compounds that eradicated uropathogenic *E. coli* in pre-formed biofilms on silicone surfaces for 24 h at 37 °C.

Combination (mM)			ΣFIC	Interaction at the MBEC
Protocatechuic Acid	Vanillic Acid	Catechin		
5.51	3.56	13.78	0.56	Additive
3.2	2.97	1.72	0.30	Synergistic
1.62	5.94	3.44	0.43	Synergistic
-	5.94	13.78	0.38	Synergistic

**Table 5:** Antioxidant activity of single phenolic compounds determined by DPPH, ABTS, and FRAP method, and expressed as the equimolar antioxidant activity of each phenolic compound with respect to the Trolox standard.

	mmol of Trolox Equivalent/mmol of Phenolic Compound		
Phenolic Compounds	DPPH	ABTS	FRAP
PA	1.36 ± 0.008 ^b^	5.77 ± 0.34 ^b^	3.65 ± 0.03 ^b^
VA	0.15 ± 0.002 ^c^	3.45 ± 0.21 ^c^	1.96 ± 0.05 ^c^
Cat	2.78 ± 0.01 ^a^	11.32 ± 0.2 ^a^	3.86 ± 0.004 ^a^

Mean ± standard deviation. PA = protocatechuic acid, VA = vanillic acid, Cat = catechin. Different letters indicate significant differences among treatments per method (*p* ≤ 0.05).

**Table 6:** Experimental antioxidant activity of the combined phenolic compounds and the differences with the expected theoretical values, considering the addition of the antioxidant capacity of the individual compounds. The used concentrations of the ternary combinations were those found previously as MIC/MBIC and MBEC.

Phenolic Combination	DPPH^•^ (µM TE)	Difference in DPPH *	ABTS (µM TE)	Difference in ABTS*	FRAP (µM TE)	Difference in FRAP *
MIC/MBIC ^a^	236.48 ± 1.41	141.11	599.0 ± 8.69	134.65	307.24 ± 3.43	9.48
MBEC ^b^	413.12 ± 2.40	30.60	1342.32 ±62.6	−213.53	1242.95 ±24.4	416.06

* Difference represents the subtraction of the experimental antioxidant capacity value for the combination minus the expected theoretical value (considered as the addition of the experimental values of the individual compounds). ^a^ MIC/MBIC = 1.62 mM protocatechuic acid + 0.74 mM vanillic acid + 0.05 mM catechin (this combination was diluted 25 times to fit the Trolox standard curves). ^b^ MBEC = 3.20 mM protocatechuic acid + 2.97 mM vanillic acid + 1.72 mM catechin (this combination was diluted 25 times to fit the Trolox standard curves). Theoretical antioxidant capacity for MIC/MBIC _DPPH_ = 95.37 µM TE, MIC/MBIC _ABTS_ = 394.35, µM TE MIC/MBIC _FRAP_ = 297.77 µM TE, MEBC _DPPH_ = 382.52 µM TE, MEBC_ABTS_ = 1555.85 µM TE, MEBC_FRAP_ = 826.9 µM TE. Mean ± standard deviation. TE = Trolox Equivalents, PA = protocatechuic acid, VA = vanillic acid, Cat = catechin.

## Supplementary Materials

The following are available online. Figure S1: Uropathogenic *Escherichia coli* growth curve at 37 °C inoculated in LB broth.
