# Supplementary material for: Comparison of Single and Combined Use of Catechin, Protocatechuic, and Vanillic Acids as Antioxidant and Antibacterial Agents against Uropathogenic Escherichia Coli at Planktonic and Biofilm Levels
Source: Molecules. 2018 Oct 30;23(11):2813. doi: 10.3390/molecules23112813 (PMC6278301; doi:10.3390/molecules23112813)

Comparison of Single and Combined Use of Catechin, Protocatechuic, and Vanillic Acids as Antioxidant and Antibacterial Agents Against Uropathogenic *Escherichia Coli* at Planktonic and Biofilm Levels

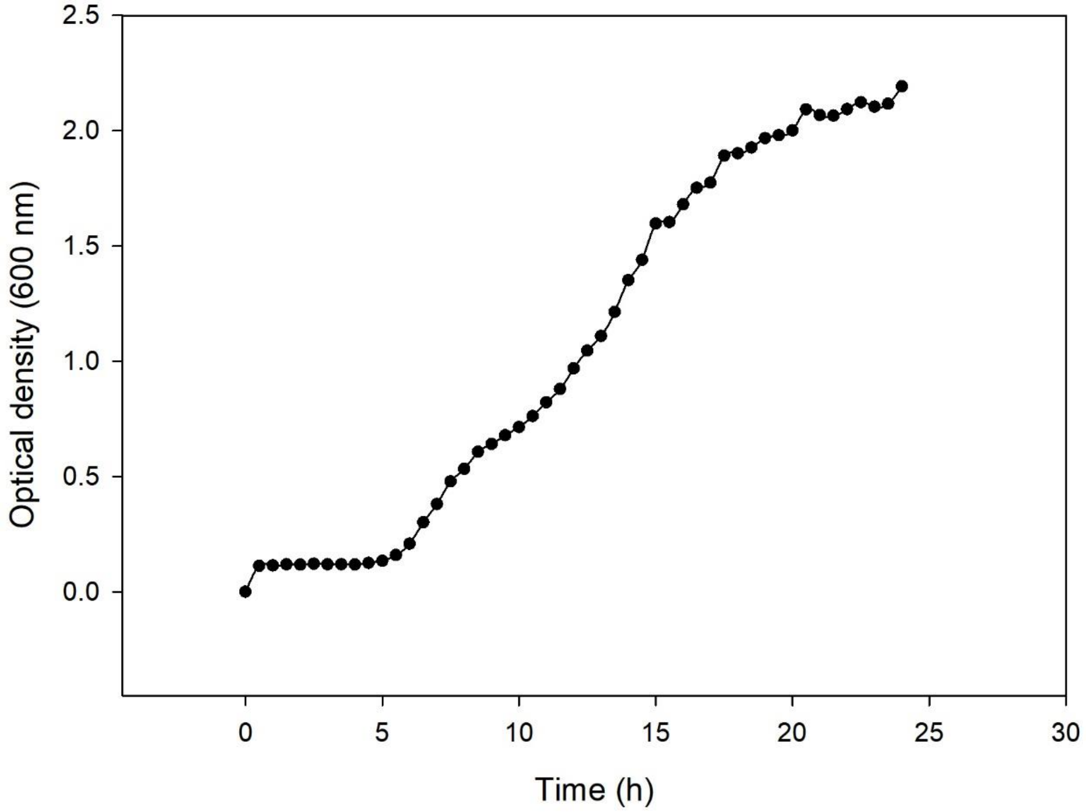

Supplement: Supplementary file 1 [file molecules-23-02813-s001.pdf]
